# Analyses of *Leishmania*-LRV Co-Phylogenetic Patterns and Evolutionary Variability of Viral Proteins

**DOI:** 10.3390/v13112305

**Published:** 2021-11-19

**Authors:** Alexei Y. Kostygov, Danyil Grybchuk, Yulia Kleschenko, Daniil S. Chistyakov, Alexander N. Lukashev, Evgeny S. Gerasimov, Vyacheslav Yurchenko

**Affiliations:** 1 Life Science Research Centre, Faculty of Science, University of Ostrava, 71000 Ostrava, Czech Republic; danilaman@gmail.com; 2 Zoological Institute of the Russian Academy of Sciences, 199034 St. Petersburg, Russia; 3 Central European Institute of Technology, Masaryk University, 60177 Brno, Czech Republic; 4 Martsinovsky Institute of Medical Parasitology, Sechenov University, 119435 Moscow, Russia; ykleschenko@gmail.com (Y.K.); dchistakoff@gmail.com (D.S.C.); alexander_lukashev@hotmail.com (A.N.L.); 5 Faculty of Bioengineering and Bioinformatics, Lomonosov Moscow State University, 119991 Moscow, Russia; 6 Faculty of Biology, M.V. Lomonosov Moscow State University, 119991 Moscow, Russia; jalgard@gmail.com; 7 Institute for Information Transmission Problems, Russian Academy of Sciences, 127051 Moscow, Russia

**Keywords:** *Leishmaniavirus*, coevolution, phylogenomics

## Abstract

*Leishmania* spp. are important pathogens causing a vector-borne disease with a broad range of clinical manifestations from self-healing ulcers to the life-threatening visceral forms. Presence of *Leishmania RNA virus* (LRV) confers survival advantage to these parasites by suppressing anti-leishmanial immunity in the vertebrate host. The two viral species, LRV1 and LRV2 infect species of the subgenera *Viannia and Leishmania,* respectively. In this work we investigated co-phylogenetic patterns of leishmaniae and their viruses on a small scale (LRV2 in *L. major*) and demonstrated their predominant coevolution, occasionally broken by intraspecific host switches. Our analysis of the two viral genes, encoding the capsid and RNA-dependent RNA polymerase (RDRP), revealed them to be under the pressure of purifying selection, which was considerably stronger for the former gene across the whole tree. The selective pressure also differs between the LRV clades and correlates with the frequency of interspecific host switches. In addition, using experimental (capsid) and predicted (RDRP) models we demonstrated that the evolutionary variability across the structure is strikingly different in these two viral proteins.

## 1. Introduction

The parasitic flagellates of the genus *Leishmania* (Trypanosomatidae) are causative agents of leishmaniasis, a vector-borne disease with a broad range of clinical manifestations from self-healing ulcers to the life-threatening visceral form [1,2]. These protists have been documented to bear negative-sense single-stranded RNA viruses of the family *Leishbunyaviridae* and double-stranded RNA (dsRNA) viruses of the family *Totiviridae* [3,4]. The first group appears to be specific to Trypanosomatidae and its members have been found in various lineages of these flagellates [5]. In *Leishmania*, the only known representative of bunyaviruses is *Leishmania martiniquensis leishbunyavirus 1* (*Lmar*LBV1), inhabiting the corresponding species of the subgenus *Mundinia* and marginally increasing the infectivity of the flagellate host in vitro [4]. *Totiviridae* are generally known as myco-viruses since most members infect fungi [6]. Other known hosts are animals [7,8,9,10] and protists [11,12,13,14]. *Leishmania RNA virus* (LRV) was first described from *Leishmania* spp. [15,16]. The two species, LRV1 and LRV2 have been documented as infecting members of the subgenera *Viannia* (*L. braziliensis*, *L. guyanensis*, *L. lainsoni*, *L. naiffi*, *L. panamensis*, and *L. shawi*) and *Leishmania* (*L. aethiopica*, *L. infantum*, *L. major*, *L. tropica*), respectively [17]. Recently, two additional species, LRV3 and LRV4, were discovered in the flea-infecting trypanosomatids of the genus *Blechomonas* [18]. LRVs confer survival advantage to *Leishmania* by suppressing anti-leishmanial immunity in the vertebrate host [19,20]. It has been experimentally demonstrated that dsRNA of LRV1 interacts with TLR3 endosomal receptor, thereby promoting chronic inflammation and mediating the spread of *L. guyanensis* to secondary sites, ultimately causing persistent “metastatic” infection [21,22,23]. Similarly, presence of LRV2 in *L. aethiopica* causes elevated levels of pro-inflammatory cytokines in murine macrophages in vitro [24]. It has been demonstrated that activation of TLR3 by viral dsRNA also leads to phosphorylation of a protein kinase B (Akt) facilitating survival and proliferation of infected macrophages [25]. It is generally assumed that these mechanisms are responsible for mutualistic co-evolution of *Leishmania* and LRV [26].

Despite the advantage provided by LRVs to *Leishmania*, they are not present in all species or isolates of a given species, suggesting more complex relationships between the two partners of this symbiosis [27]. Moreover, it appears that the presence of viruses in some *Leishmania* spp., for example LRV2 in *L. tropica* and *L. infantum*, can be explained by the relatively recent horizontal transfers [28,29]. This agrees with the experimental data on the possibility of LRV transmission via exosomes, which are shed by the infected leishmaniae and, subsequently, uptaken by uninfected ones through the flagellar pocket [30].

In this work we attempted to shed light on the evolution of LRVs by investigating co-phylogenetic patterns of leishmaniae and their viruses on a small scale, as well as scrutinizing the evolutionary variability of the two viral proteins, the capsid and reverse RNA-dependent RNA polymerase (RDRP).

## 2. Materials and Methods

### 2.1. Leishmania spp. Strains, Cultivation, and Screening for dsRNA Viruses

Promastigotes of 21 *Leishmania* spp. isolates (Table 1) were cultivated as described previously [31]. Total genomic DNA was isolated from 10 mL of the log-phase cultures using DNeasy Blood & Tissue Kit (Qiagen, Hilden, Germany) according to the manufacturer’s instructions. Small subunit rRNA gene was amplified using primers S762 and S763, following the previously described protocol [32] and sequenced directly at Macrogen Europe (Amsterdam, The Netherlands) with a set of internal primers as reported elsewhere [33]. The species identity of the strains was confirmed by BLAST. Double-stranded RNA fractions were isolated and analyzed as described previously [5]. The presence of LRVs in the strains MHOM/UZ/1998/IsvM01h and MHOM/UZ/1998/IsvM27h (Table 1) and viral sequences have been reported earlier [34]. In this work we sequenced the genomes of these two *Leishmania* isolates.

For the comparison of LRV2 prevalence, 95% confidence interval (CI) was calculated by “exact” binomial method (Clopper–Pearson interval), and the statistical significance of the difference in the observed proportions has been estimated using the chi-square test.

### 2.2. Whole-Genome Sequencing and Read Mapping

Total DNA of 9 virus-positive and 1 virus-negative (13 Th) *L. major* strains was isolated as described previously [35]. The whole-genome sequencing libraries were prepared with TruSeq DNA PCR-free Kit (Illumina, San Diego, CA, US) and sequenced using HiSeq 4000 platform (Illumina) in paired-end mode with read length of 150 bp at SkolTech Genomics core facility (Moscow, Russia). The average sequencing depth was approximately 27 million read pairs per sample. The obtained data were deposited to GenBank (BioProject PRJNA763936).

Read quality control was performed with FastQC v. 0.11.9 [36]. Reads were adapter- and quality-trimmed with Trimmomatic v. 0.39 [37] and mapped on the reference genome assembly of *Leishmania major* strain Friedlin from TriTrypDB v. 50 [38] using BWA-MEM algorithm in BWA v. 0.7.17 [39].

Isolated double-stranded RNA samples were sequenced at Macrogen (Seoul, Korea) and the obtained raw reads were used for assembling viral genomes as described previously [18]. The assembled sequences were deposited to GenBank under the accession numbers MZ926700-MZ926706.

### 2.3. Phylogenetic Analyses of Leishmania major Strains

In addition to 10 *L. major* strains studied here, whole-genome sequencing data for the following strains: SD75, LV39c5, LT252, BH129 and BH121 were downloaded from the NCBI SRA database (accession numbers SRR833759, SRR1028158, ERR3610774, SRR14328165, SRR14328166, respectively). Reads were processed and mapped as described above, but specific trimming parameters were chosen individually according to the FastQC reports. The genome assembly and annotation of *L. aethiopica* isolate L147 was downloaded from the TriTrypDB. One-to-one orthologs (202 genes in total) between *Leishmania major* and *L. aethiopica* were found using the NCBI BLAST suite v. 2.12.0+ [40].

The single nucleotide polymorphism (SNP) calling was done with GATK v. 4 [41]. The vcf-consensus program from the VCFtools v. 0.1.16. package [42] was used to coordinate SNPs with the reference genome of *L. major* strain Friedlin and to obtain strain-specific sequences for further multiple sequence alignments. Individual SNPs, showing no variation between the strains sequenced here, were filtered out.

Multiple sequence alignment was performed using MAFFT v. 7.475 using L-INS-i algorithm [43]. Phylogenetic tree was constructed using IQ-Tree v. 1.6.12 [44] with best-fit model (TIM + F + G4) determined by ModelFinder [45] and testing tree branches by 1000 standard bootstraps replicates.

### 2.4. Phylogenetic Analyses of LRVs

In addition to the sequences obtained in this work, all available genomes of LRVs were retrieved from the NCBI (Table S1). Codons were determined and translated using OrfM v. 0.7.1 [46]. Amino acid sequences were aligned with MAFFT v. 7.453 using E-INS-i algorithm and trimmed with trimAl v. 1.4 [47] using “-colnumbering” option to keep track of deleted columns. The amino acids in the trimmed alignment were reverted to the corresponding codons using a custom script. The resulting nucleotide alignment was split into three partitions according to the codon position using a custom script in conjunction with the trimAl “-selectcols” option. The substitution model was selected separately for each position using the ModelFinder: GTR + F + G4 for the 1st and 2nd positions and GTR + F + I + G4 for the 3rd position. The maximum likelihood analysis was performed in IQ-Tree v. 1.6.12 with unlinked branch lengths across partitions and 1000 thorough bootstraps replicates.

To assess the mode of sequence evolution, the codon-based z-test for selection was conducted in MEGA X [48,49] using the modified Nei-Gojobori method [50] with transition/transversion ratio of 2 and 1000 bootstrap replicates to calculate the variance of the difference. This analysis involved 35 LRV1 and 13 LRV2 nucleotide sequences. All ambiguous positions were removed for each sequence pair (“pairwise deletion” option) giving a total of 1586 (LRV1) and 1556 (LRV2) codon positions in the final dataset. The omega values (dN/dS ratio) for each protein alignment have been estimated using SLR v. 1.3 [51].

### 2.5. Structure Prediction and Functional Sites Annotation

The structure of LRV1 capsid protein and functional sites have been already experimentally determined [52]. No such information is available for RDRP of LRVs and, therefore, its secondary and tertiary structures have been predicted using AlphaFold 2 [53] with a “full_dbs” preset and visualized in UCSF ChimeraX v. 1.2.5 [54]. Annotation of functional elements has been performed manually based on the data from [55]. Alignments were visualized in Jalview v. 2.11 [56].

## 3. Results

### 3.1. Screening of Leishmania spp. Isolates for the Presence of dsRNA Viruses

Nineteen isolates of *Leishmania* spp. from Middle Asia (twelve from Uzbekistan and seven from Turkmenistan) were unambiguously identified as *L. major* or *L. turanica* using 18S rRNA gene sequences and screened for the presence of leishmaniaviruses (Table 1). For *L. major*, seven out of eleven strains were virus-positive, while all eight *L. turanica* isolates were virus-negative. Combined with the previous report on *Leishmania* spp. strains from the same area [34], LRV2 prevalence is 0% (0/12; 95% CI: 0–26.5%) in *L. turanica* and 64.3% (9/14; 95% CI: 35.1–87.2%) in *L. major* with the difference well supported by the chi-square test (*p* = 0.0008). Although the viruses appeared to be more frequent in the *L. major* isolates from humans (70%, 7/10; 95% CI: 34.7–93.3%) than in those from great gerbils (50%, 2/4; 95% CI: 6.8–93.2%), this could not be confirmed statistically (*p* = 0.5).

### 3.2. Phylogenetic Analyses of Leishmania major Strains and Their Viruses

**Figure 1:**
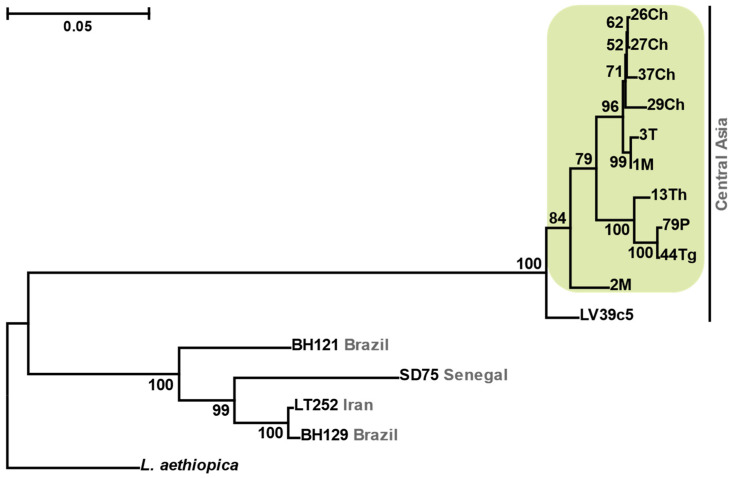
Maximum likelihood phylogenetic tree of *L. major* isolates inferred using SNP data. Scale bar indicates the number of substitutions per site. Values at branches are bootstrap supports. *Leishmania aethiopica* was used as an outgroup. Country/region of origin is specified in grey. Isolates studied here are highlighted in green.

The phylogenetic analysis based on SNP data for 202 genes from the available *L. major* genomes demonstrated that all 10 strains studied here are monophyletic (Figure 1), agreeing with their common geographic origin from Uzbekistan. The same concerns the strain LV39, isolated in an unspecified Central Asian region of the former Soviet Union and adjoining this clade. Meanwhile, the remaining isolates, collected in other parts of the world (Iran, Senegal, and Brazil), are separated from these by a long branch.

**Figure 2:**
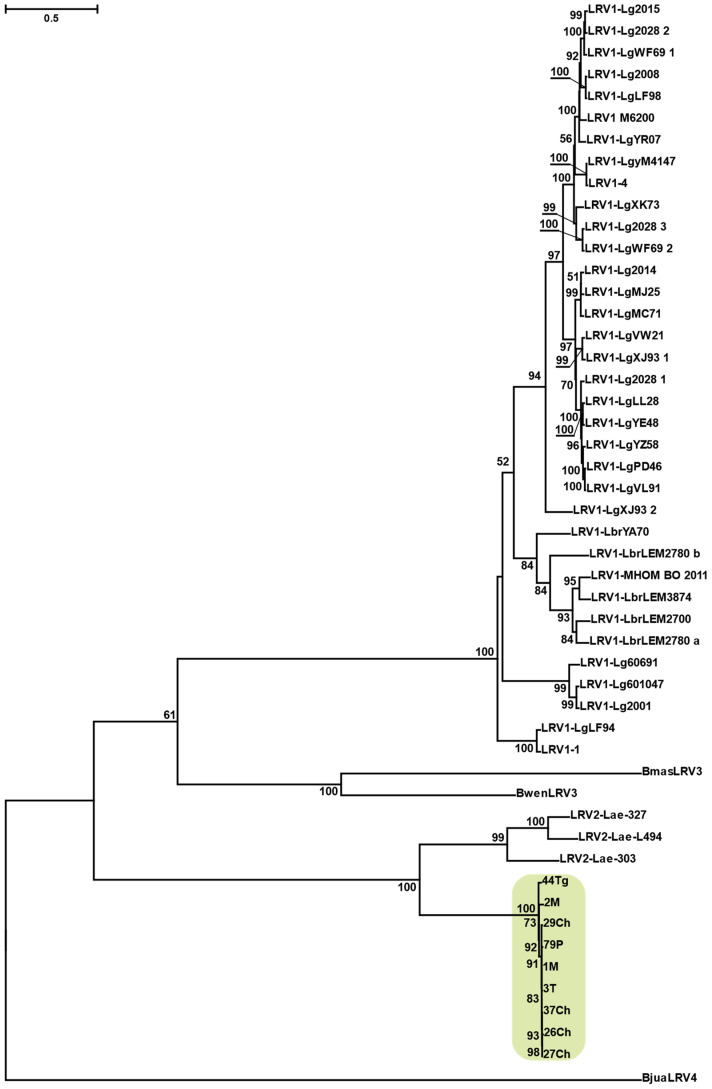
Maximum likelihood phylogenetic tree of leishmaniaviruses inferred using the concatenated nucleotide alignments of RDRP and capsid genes. Scale bar indicates the number of substitutions per site (please note that the evolutionary rates between different codon positions are extremely different, explaining the inferred lengths of some branches over 1). Values at branches are bootstrap supports. Isolates studied here are highlighted in green. See Table S1 for accession numbers of sequences retrieved from the NCBI.

The topologies of the inferred RDRP and capsid phylogenetic trees were generally similar (Figures S1 and S2) with small differences in some poorly resolved parts (for example, viruses from *Blechomonas*). Therefore, it was possible to concatenate the two codon-based nucleotide alignments and obtain a more robust phylogenetic tree (Figure 2), where, in particular, the position of *Blechomonas* LRVs coincided with that, previously inferred using amino acid sequences [18]. Similarly to their trypanosomatid hosts, the viruses from *L. major* strains analyzed here also formed a monophyletic group. The phylogenetic distances within this group were very short, as compared to the rest of the tree. The LRV2 from *L. aethiopica* also proved to be monophyletic, whereas the LRV1 viruses from *L. braziliensis* and *L. guyanensis* were intermingled (Figure 2) demonstrating an extensive viral exchange between these two species.

**Figure 3:**
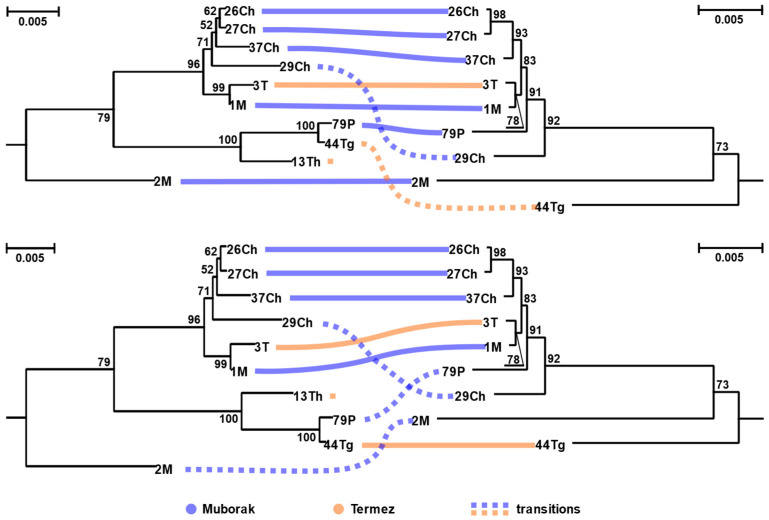
Juxtaposition of subtrees for *Leishmania major* and the corresponding LRV2 clade demonstrating the two most parsimonious scenarios of coevolution and host switches. Scale bar indicates the number of substitutions per site. Values at branches are bootstrap supports.

The monophyly of *L. major* from Uzbekistan and that of their viruses prompted us to investigate their co-phylogenetic patterns. The topologies within the corresponding clades were similar, but not fully congruent, suggesting predominant coevolution, occasionally broken by intraspecific host switches (Figure 3). Such switches should have occurred at least twice: (i) in the isolate 29Ch and (ii) either in 44Tg or 79P (Figure 3, top and bottom panels, correspondingly). Due to uncertain position of the isolate 2M on both trees, the exact number of host switches (2 or 3) in either scenario cannot be established with certainty. There was no correlation between tree topologies and the geographic origin of the isolates. This is consistent with the fact that distance between the two sampling locations (Termez and Muborak) is only ~290 km, which is not long enough to create isolated populations of *L. major*, a parasite with highly mobile hosts and vectors.

### 3.3. Mode of RDRP and Capsid Genes Evolution

**Figure 4:**
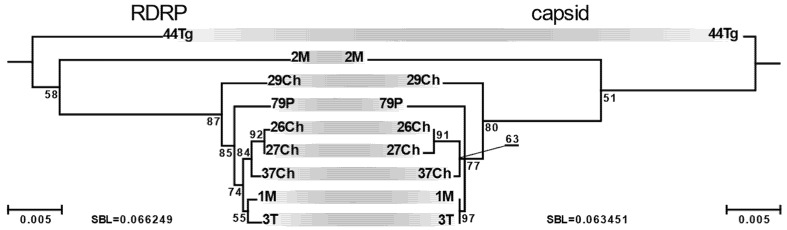
Comparison of RDRP and capsid subtrees for the LRV2 from *L. major* clade drawn to the same scale. SBL stands for the sum of branch lengths. Scale bar indicates the number of substitutions per site. Values at branches are bootstrap supports.

In general, the two viral genes appear to evolve similarly. Considering the clade of LRV2 from *L. major* (Figure 2), we observed not only collinear topologies, but also similar rates of evolution as judged by the sums of branch lengths, which for the capsid is ~0.95 of that for the RDRP (Figure 4). However, it is evident that the lengths of individual branches in the capsid subtree are heterogeneous, while the RDRP subtree is almost ultra-metric. Given that both genes locate on a single genomic segment and evolve at approximately the same rate, we posited that this discrepancy may be explained by different (and variable) levels of selective pressure. The *z*-test of selection revealed statistically significant deviation from the strict neutrality in favor of negative (purifying) selection, both in the whole LRV dataset, as well as in the subset of LRV2 from *L. major* (*p* < 10^−10^). In a pairwise mode, the results of the tests were similar, except for a few cases of closely related sequences, where the number of substitutions was apparently insufficient for calculations (Table S2).

In order to understand the differences in the selective pressure on both genes, we estimated the omega (dN/dS) values, i.e., ratios of nonsynonymous to synonymous substitutions in both genes for the whole tree and particular clades (Table 2). The results demonstrate that nonsynonymous substitutions are approximately three–four times less frequent in the capsid as compared to the RDRP, indicating that the effect of the purifying selection is considerably stronger in the former. For example, in the LRV2 from *L. major* 32/151 (21.2%) variable sites of RDRP contained nonsynonymous substitutions, while this ratio for the capsid was only 9/120 (7.5%). Counterintuitively, the omega values estimated for both genes in LRV2 from *L. major* were considerably higher than in other clades, especially in the sister phylogroup of LRV2 from *L. aethiopica*, or in the whole tree (Table 2). One would expect higher estimated dN/dS ratios at larger distances, since more frequent synonymous substitutions in these conditions can be underestimated due to the mutational saturation. The opposite results observed here indicate non-homogeneity of selection across the clades on the tree.

### 3.4. Sequence Conservation of LRV Proteins across the Structure

Since the studied genes encode essential proteins, their evolution must be inevitably driven by functional constraints. Hence, we decided to investigate how the structure of these proteins correlates with their sequence conservation in order to understand why the selective pressure on these two genes is different.

#### 3.4.1. Capsid

We used the recently published data on the experimentally determined structure of LRV1 capsid [52]. It was shown that two differently folded copies of the protein form an icosahedral asymmetric unit of the viral particle. As in the related *Saccharomyces cerevisiae virus L-A*, the bulk of the capsid protein is composed of alpha-helices, while the less abundant beta-sheets face toward the two-fold and three-fold symmetry axes and provide interaction surfaces between capsid proteins within a particle [57].

**Figure 5:**
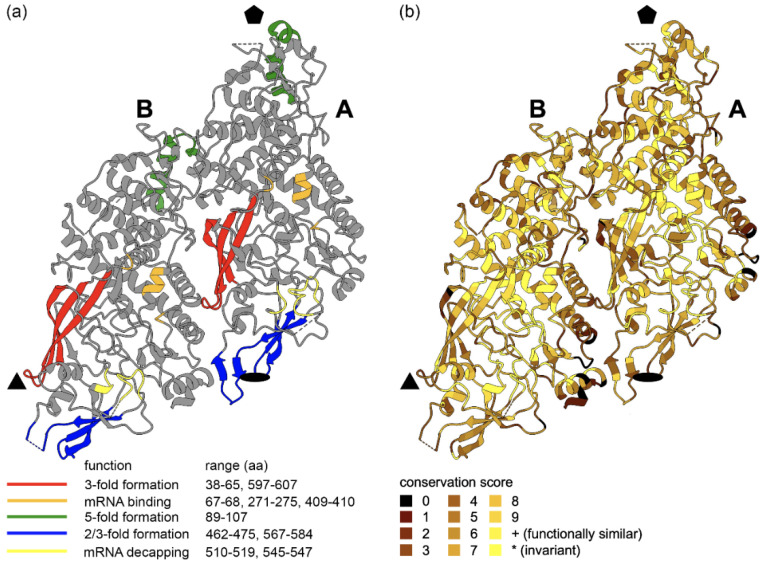
Three-dimensional structure of LRV1 capsid protein showing functional motifs and conservation. The asymmetric capsid unit, formed by subunits A and B, representing folding variants of the same protein, is shown. The approximate positions of five-fold, three-fold and two-fold symmetry axes around the unit are marked by black pentagon, triangle and ellipse, respectively. (**a**) Position of functional regions (residue numbering is for LRV1-LgyM4147). (**b**) Conservation of amino acid residues in the alignment (Figure S3). Conservation scores are calculated in Jalview.

Within both variants of the capsid protein, internal alpha-helices and loops involved in host mRNA binding and decapping demonstrate high degree of sequence conservation (Figure 5). At the same time, flexible loops between major secondary structure elements display more sequence variability. Beta-sheets in the lower left portion of the capsid protein (red and blue in Figure 5a) form interaction surfaces between units (at two- and three-fold symmetry axes) and between A and B subunits of a unit. They demonstrate intermediate overall conservation, although within a particular LRV species the sequences are highly similar (Figure S3). The helix-turn-helix motif (green in Figure 5a) is essential for the formation of five-fold symmetry axes of the virus particle. The mRNA binding and mRNA decapping motifs (orange and yellow in Figure 5a, respectively) are largely conserved (except for Arg68), but devoid of secondary structures. Unexpectedly, the residues outlining pores, which are necessary for the exit of viral RNA to enable translation, as well as the entry of nucleotides for replication, demonstrate conservation only within a particular LRV species, sometimes differing even between the two clades of LRV2 (Figure S3). Thus, efficient folding and virion assembly, together with host mRNA decapping, represent important constraints in the evolution of LRV capsid protein. However, the high conservation of some alpha helices with no functional annotation (Figure 5b and Figure S3) indicates that the capsid protein may contain additional crucially important components.

#### 3.4.2. RDRP

**Figure 6:**
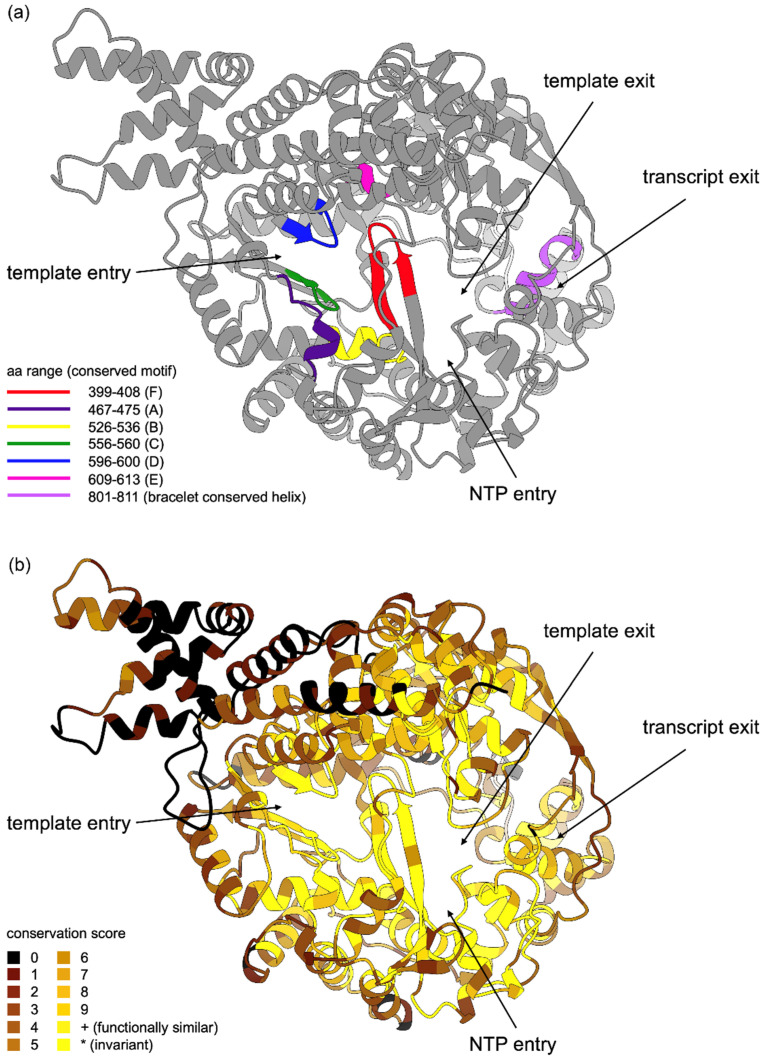
Predicted three-dimensional structure of RDRP protein in LRV1 demonstrating functional motifs and conservation. (**a**) Position of functional regions (residue numbering is for LRV1-LgyM4147). Unannotated parts of the molecule are in grey. (**b**) Conservation of amino acid residues in the alignment (Figure S4). Conservation scores are calculated in Jalview. Distant parts of the molecule are shown with lighter shades of colors.

Since the RDRP structure was never determined experimentally for any of the LRVs, we employed AlphaFold 2 to predict it. According to the inferred model (Figure 6), the polymerase has a typical enclosed “right-hand” architecture forming a passage for viral RNA and is structurally similar to that of picorna- and caliciviruses (PDB accession numbers 1wne, 2ijd, 1sh0, 3uqs).

The inner residues of RDRP forming the catalytic core and RNA channel are highly conserved (Figure 6). The outer surface of the polymerase shows moderate conservation, whereas the N-terminal part (residues 1–158) is largely variable. To test whether such variability is associated with changes in selective pressure, we applied the *z*-test to the 120 N-terminal codons in the alignment. Our results indicated positive selection for the most sequence pairs combining LRV2 from *L. aethiopica* and LRV1 (Table S3). The template entry is outlined by residues 254–308 and immediately leads to the polymerase catalytic core formed by six structural motifs A-F [55], which are invariant in all leishmaniaviruses (Figure S4). Positively charged residues in the motifs F (Lys399, Lys404 and Arg406) and D (Arg599), as well as Asp469 in the motif A, are crucial for NTP selection and binding, whereas negatively charged aspartates in the motifs A (Asp467) and C (Asp558) are important for the Mg^2+^ ions coordination and catalysis [58]. The RNA channel within the polymerase is split into two paths by the alpha helix 801–811, which is largely conserved in all leishmaniaviruses. We hypothesize that this helix facilitates RNA strand separation similarly to the bracelet domain of cytoplasmic polyhedrosis virus [59] and rotavirus [60]. Indeed, the surface of this helix is negatively charged creating a steric obstacle for the incoming RNA duplex and directing the strands into two separate positively charged channels. In this process, the hydrophobic patch (residues 803–807) is important for breaking the hydrogen bonds between RNA bases, while positively charged outlines of the exit channels capture the phosphate groups of the RNA backbone [60]. The strand separation is important for all dsRNA viruses since transcript sense RNA must exit virion through pores for translation and new particle assembly [59,61].

## 4. Discussion

Here we report a high prevalence (over 60%) of LRV2 in *Leishmania major*, similar to that previously recorded in Northern Iran (~70%) [29], which is situated approximately 1000 km from the area sampled in this work. No viral infection was detected in *L. turanica*, one of the closest relatives of *L. major* [62,63] coinfecting the same sand fly vector, *Phlebotomus papatasi*, or the great gerbils [64,65]. This is particularly surprising, given that more distantly related *L. tropica* and *L. infantum* bear LRVs obtained by horizontal transfer from *L. major* [28,29,66]. The viruses inhabiting *L. aethiopica*, the sister species of *L. tropica*, represent a separate LRV2 lineage (Figure 2), which apparently evolved in isolation, since the geographic range of its flagellate host (Ethiopia and Kenya highlands) does not overlap with those of abovementioned *Leishmania* spp. [67].

Even with a relatively small number of strains we were able to reveal intraspecific horizontal transfers of LRV2 between *L. major* strains, superimposed on their co-phylogenetic pattern. The observed results are consistent with the paradigm that, similarly to many other parasitic protists, *Leishmania* spp. reproduce mostly clonally [68], but occasionally different strains or species meet and exchange their genetic material and/or viruses [17,69,70,71]. The transfer of LRVs does not even require a direct cell contact—the transmission is efficiently completed via exosomes shed and subsequently uptaken through the flagellar pocket [30]. If the viral prevalence is high, this may lead to a superinfection of a single *Leishmania* isolate with several LRV strains [72], but in this study we did not detect such cases.

The clade of LRV2 from *L. major* evolves differently from other LRVs, as judged by a higher ratio of nonsynonymous to synonymous substitutions. We hypothesize that the promiscuity of this lineage (ability to infect other *Leishmania* spp.) results in the lowered selective pressure for this virus, since life in different flagellate host species may weaken the stabilizing selection. This effect is not observed in LRV1, which also switches between different species (*L. guyanensis* and *L. braziliensis*), but its hosts are more closely related. In addition, the whole history of LRV1 was tightly associated with these *Leishmania* spp. resulting in the adaptation to the regular host switches. Of note, the hosts of LRV1 (subgenus *Viannia*) possess the RNAi pathway, which has been lost in the subgenus *Leishmania* hosting LRV2 [73,74]. It may influence the selective pressure on LRVs, although we did not observe a strict correlation between RNAi presence and omega values for the capsid and RDRP in different lineages.

The comparison of the two viral genes revealed that their evolutionary constraints differ, with RDRP being more permissive to nonsynonymous substitutions. This can be explained by drastically different structural-functional properties of these two proteins. While RDRP, as an enzyme, requires the conservation mostly in its central part associated with substrate uptake and catalytic activity, the capsid, as a multipurpose protein, contains more functional fragments and, therefore, a higher proportion of the sequence is conserved at the amino acid level.

Although in general the evolution of the two proteins appears to be under a strong purifying selection, it is remarkable that some variable regions, such as the N-terminus of RDRP and pore-associated residues, experience a selective pressure in the opposite direction, suggesting their important roles in adaptation to different hosts.

Our results allowed correlating the modes of evolution in LRVs with their biological peculiarities. We envision that further research will be focused on scrutinizing the specificity and adaptation of viruses to various hosts, and the functional analysis of uncharacterized parts of the viral proteins.

## Figures and Tables

**Table 1:** Strains of *Leishmania* spp. investigated in this work.

Species	Code	WHO Code	Source	Origin
**Negative**				
*L. turanica*	87568	MRHO/UZ/1987/KD87568	great gerbil ^1^	Uzbekistan, Qashqadaryo reg
	9554	MRHO/TM/1995/9554		Turkmenistan, Ak bugdaý, Ahal reg.
	9562	MRHO/TM/1995/9562		
	9558	MRHO/TM/1995/9558		
	KP137	MHOM/TM/1986/KP137		Turkmenistan, Serdar, Balkan reg.
	91014	MHOM/TM/1991/91014		Turkmenistan, Tjazeel, Ahal reg.
	73 P	MHOM/UZ/2002/IsvM73g		Uzbekistan, Muborak, Qashqadaryo reg.
	9105	MRHO/TM/1991/9105		Turkmenistan, Tjazeel, Ahal reg.
*L. major*	13Th	MHOM/UZ/2003/IsvT13h	human	Uzbekistan, Termez, Surxondaryo reg.
	24Th	MRHO/UZ/2003/IsvT24h		
	9537	MRHO/TM/1995/9537	great gerbil	Turkmenistan, Serahs, Ahal reg.
	BUR	MRHO/UZ/1987/BUR	human	Uzbekistan, Qorovulbozor, Bukhara reg.
**Positive**				
*L. major*	1M ^2^	MHOM/UZ/1998/IsvM01h	human	Uzbekistan, Muborak, Qashqadaryo reg.
	2M	MHOM/UZ/1998/IsvM29h		
	26Ch	MHOM/UZ/2002/IsvM26h		
	27Ch ^2^	MHOM/UZ/1998/IsvM27h		
	29Ch	MHOM/UZ/2002/IsvM29h		
	37Ch	MHOM/UZ/2002/IsvM37h		
	79P	MRHO/UZ/2002/IsvM79g	great gerbil	
	44Tg	MRHO/UZ/2003/IsvT44g		Uzbekistan, Termez, Surxondaryo reg.
	3T	MHOM/UZ/2000/IsvT03h	human	

^1^ *Rhombomys opimus* (Muridae, Rodentia). ^2^ Tested for the presence of LRV in [34].

**Table 2:** Variation of dN/dS ratio in RDRP and capsid genes across the LRV tree.

	RDRP	Capsid
LRV1	0.0617	0.0219
LRV2 from *L. major*	0.1283	0.0292
LRV2 from *L. aethiopica*	0.0455	0.0116
overall	0.0554	0.0192

## Data Availability

The sequence data obtained in this study are available from GenBank under the following accession numbers: MZ926700–MZ926706 (genomes of LRVs), SRR15924521–SRR15924530 (BioProject PRJNA763936, raw reads for *Leishmania major* genomes).

## Supplementary Materials

The following are available online at https://www.mdpi.com/article/10.3390/v13112305/s1, Figure S1: Maximum likelihood phylogenetic tree inferred using capsid sequences; Figure S2: Maximum likelihood phylogenetic tree inferred using RDRP sequences; Figure S3: Multiple sequence alignment of LRV capsid proteins; Figure S4: Multiple sequence alignment of LRV RDRP proteins; Table S1: Sequences of LRVs retrieved from Genbank; Table S2: *p*-values for the *z*-test in sequence pairs (null hypothesis—strict neutrality, alternative hypothesis—purifying selection) of capsid and RDRP proteins, Table S3: *p* values for the *z*-test in sequence pairs for the N-terminus of RDRP (both purifying and positive selection tested).
