# Supplementary figures and images for: Analyses of Leishmania-LRV Co-Phylogenetic Patterns and Evolutionary Variability of Viral Proteins
Source: Viruses. 2021 Nov 19;13(11):2305. doi: 10.3390/v13112305 (PMC8624691; doi:10.3390/v13112305)

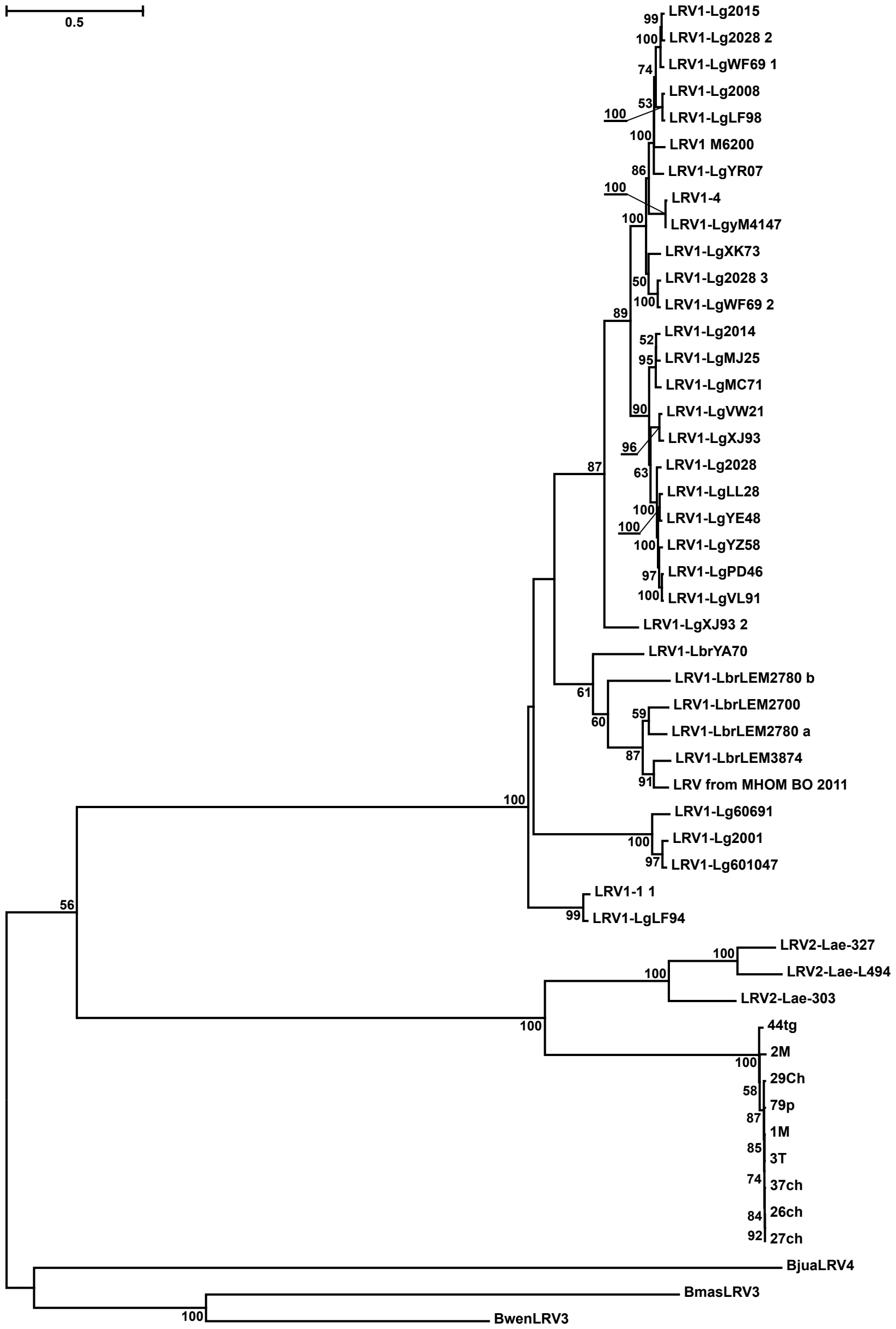

Supplement: Supplementary file 1 [file viruses-13-02305-s001.zip › Figure S1.pdf]

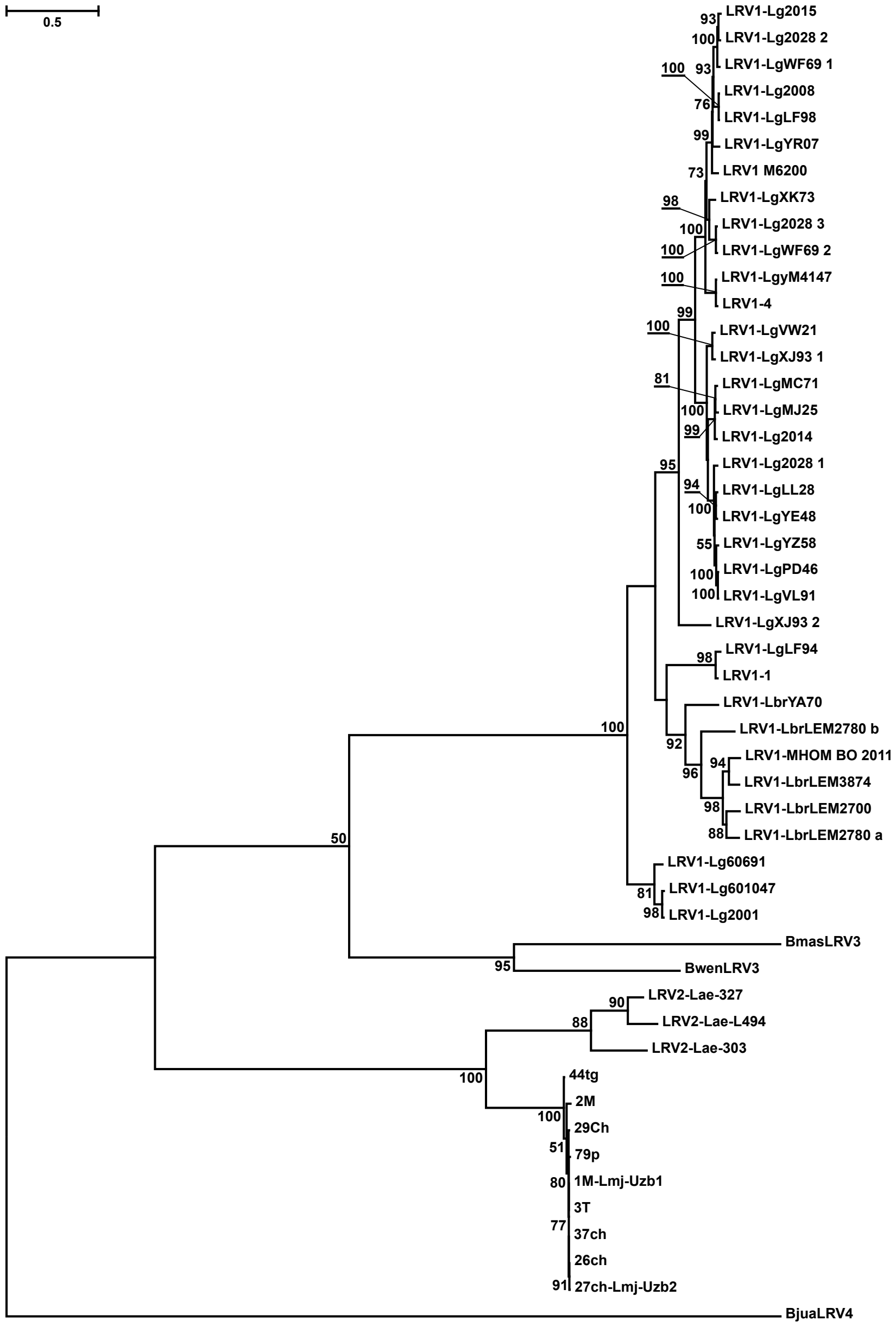

Supplement: Supplementary file 1 [file viruses-13-02305-s001.zip › Figure S2.pdf]

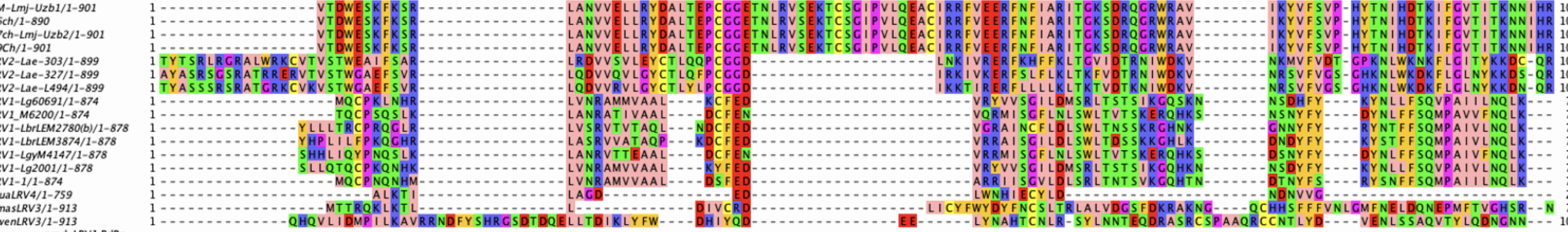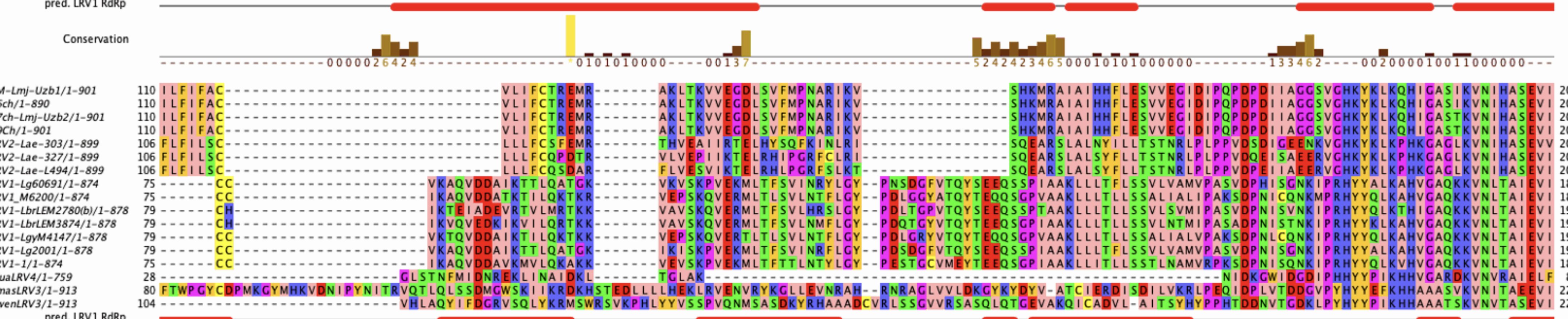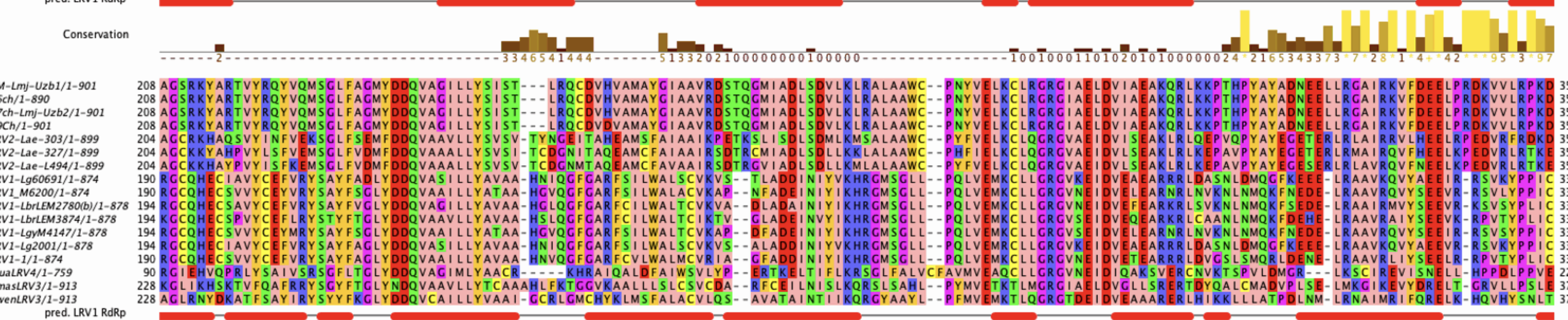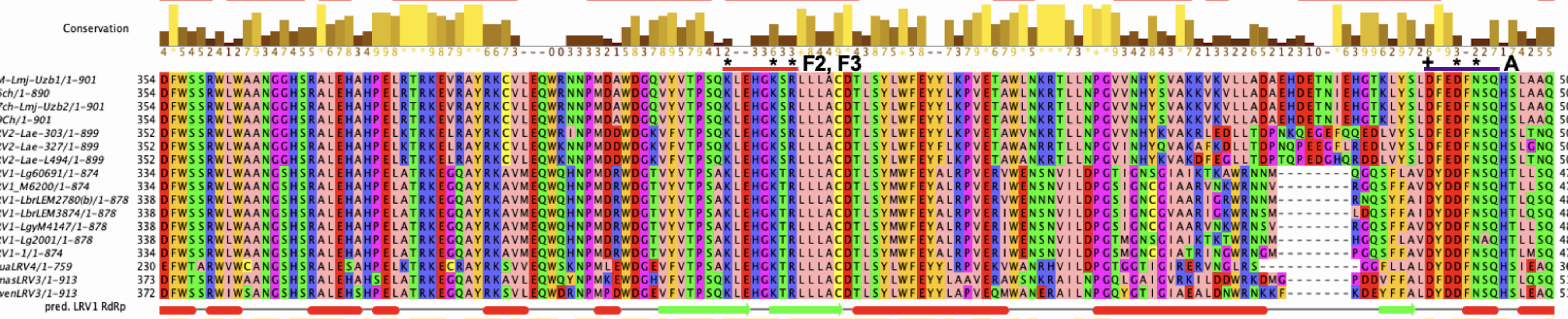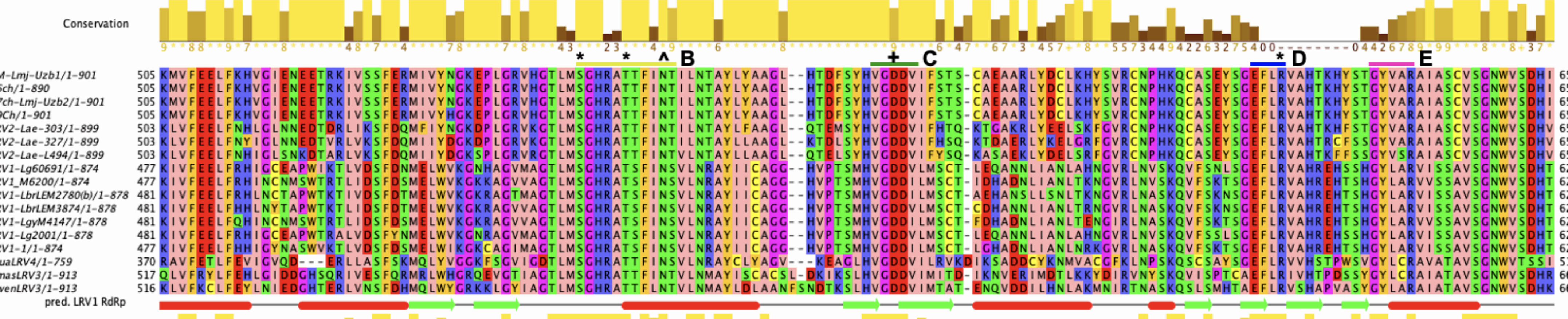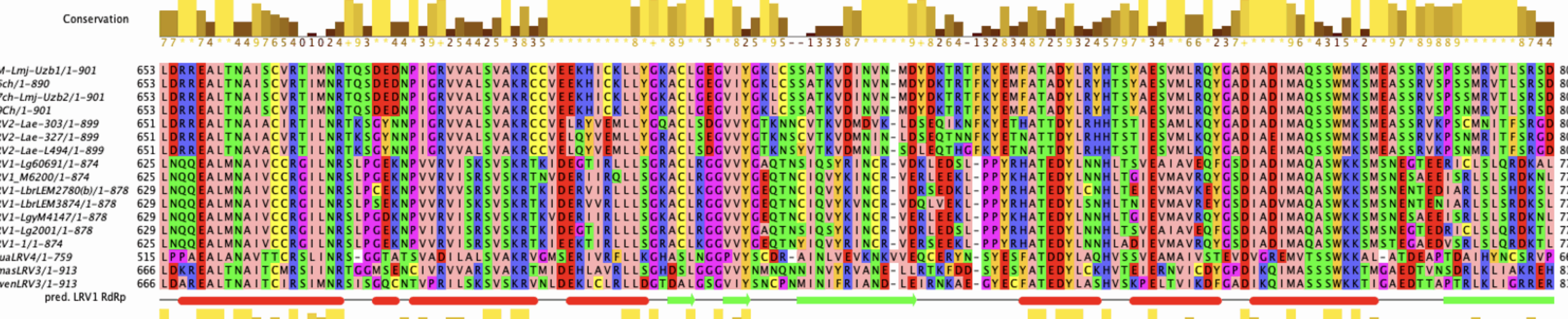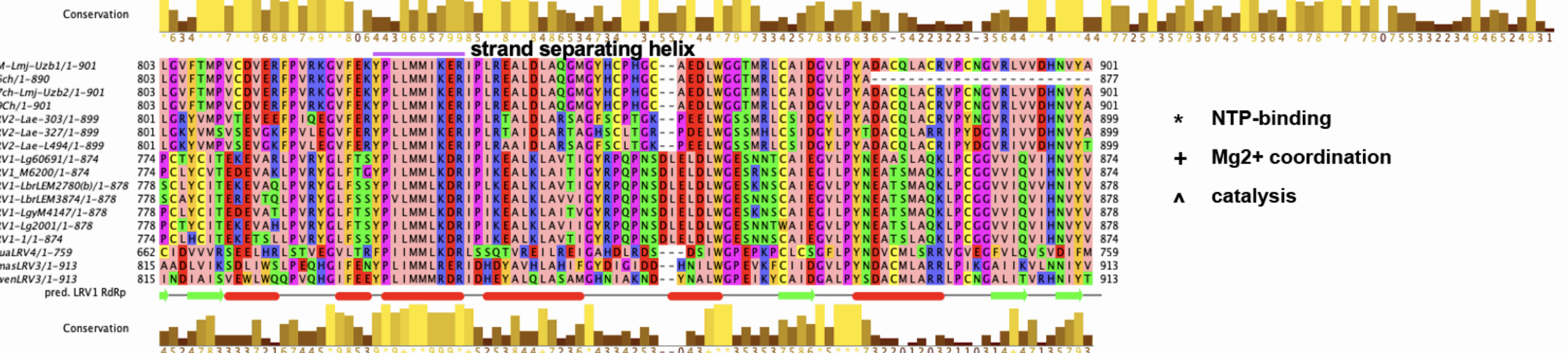

Supplement: Supplementary file 1 [file viruses-13-02305-s001.zip › Figure S4.pdf]
