# Supplementary material for: Analyses of Leishmania-LRV Co-Phylogenetic Patterns and Evolutionary Variability of Viral Proteins
Source: Viruses. 2021 Nov 19;13(11):2305. doi: 10.3390/v13112305 (PMC8624691; doi:10.3390/v13112305)
Supplement: Supplementary file 1 [file Figure S3.pdf › Figure S3.pdf]

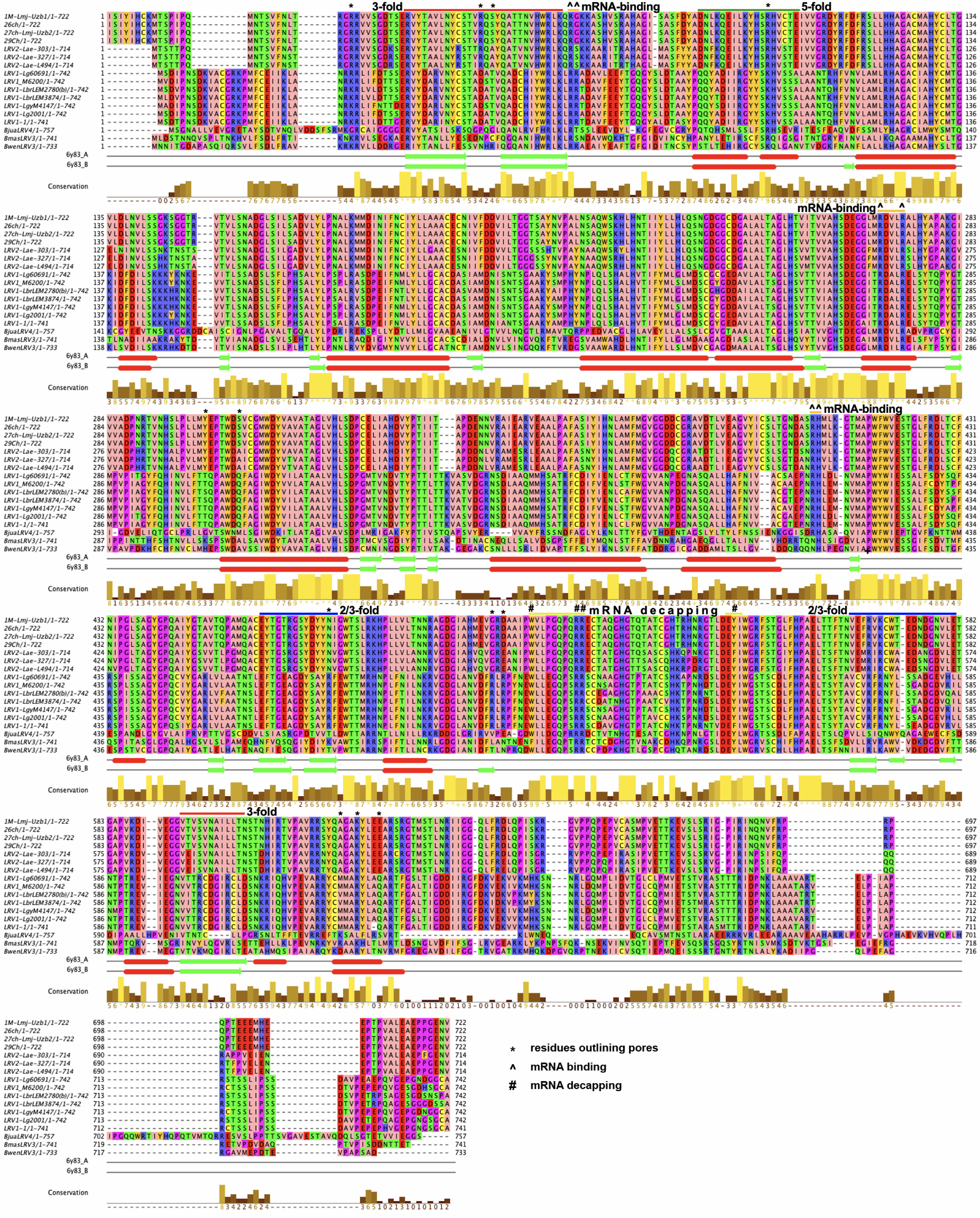

3-fold
mRNA-binding
5-fold
2/3-fold
mRNA decapping
1M-Lmj-Uzb1/1-722
26ch/1-722
27ch-Lmj-Uzb2/1-722
29Ch/1-722
LRV2-Lae-303/1-714
LRV2-Lae-327/1-714
LRV2-Lae-L494/1-714
LRV1-Lg60691/1-742
LRV1_M6200/1-742
LRV1-LbrLEM2780(b)/1-742
LRV1-LbrLEM3874/1-742
LRV1-LgyM4147/1-742
LRV1-Lg2001/1-742
LRV1-1/1-741
BjuaLRV4/1-757
BmasLRV3/1-741
BwenLRV3/1-733
6y83_A
6y83_B
Conservation
* residues outlining pores
^ mRNA binding
# mRNA decapping
